# Comparative Evaluation of Three TSPO PET Radiotracers in a LPS-Induced Model of Mild Neuroinflammation in Rats

**DOI:** 10.1007/s11307-016-0984-3

**Published:** 2016-08-01

**Authors:** Sujata Sridharan, Francois-Xavier Lepelletier, William Trigg, Samuel Banister, Tristan Reekie, Michael Kassiou, Alexander Gerhard, Rainer Hinz, Hervé Boutin

**Affiliations:** 1 Wolfson Molecular Imaging Centre, University of Manchester, 27 Palatine Road, Manchester, M20 3LJ UK; 2 GE Healthcare, The Grove Centre, Amersham, Buckinghamshire UK; 3 School of Chemistry, University of Sydney, Sydney, NSW 2006 Australia; 4 Faculty of Health Sciences, University of Sydney, Sydney, NSW 2006 Australia

**Keywords:** TSPO, Inflammation, LPS, Second-generation tracers, Preclinical kinetic modelling

## Abstract

**Purpose:**

Over the past 20 years, neuroinflammation (NI) has increasingly been recognised as having an important role in  many neurodegenerative diseases, including Alzheimer’s disease. As such, being able to image NI non-invasively in patients is critical to monitor pathological processes and potential therapies targeting neuroinflammation. The translocator protein (TSPO) has proven a reliable NI biomarker for positron emission tomography (PET) imaging. However, if TSPO imaging in acute conditions such as stroke provides strong and reliable signals, TSPO imaging in neurodegenerative diseases has proven more challenging. Here, we report results comparing the recently developed TSPO tracers [^18^F]GE-180 and [^18^F]DPA-714 with (R)-[^11^C]PK11195 in a rodent model of subtle focal inflammation.

**Procedures:**

Adult male Wistar rats were stereotactically injected with 1 μg lipopolysaccharide in the right striatum. Three days later, animals underwent a 60-min PET scan with (R)-[^11^C]PK11195 and [^18^F]GE-180 (*n* = 6) or [^18^F]DPA-714 (*n* = 6). Ten animals were scanned with either [^18^F]GE-180 (*n* = 5) or [^18^F]DPA-714 (*n* = 5) only. Kinetic analysis of PET data was performed using the simplified reference tissue model (SRTM) with a contralateral reference region or a novel data-driven input to estimate binding potential BP_ND_. Autoradiography and immunohistochemistry were performed to confirm *in vivo* results.

**Results:**

At 40–60 min post-injection, [^18^F]GE-180 dual-scanned animals showed a significantly increased core/contralateral uptake ratio *vs*. the same animals scanned with (R)-[^11^C]PK11195 (3.41 ± 1.09 *vs*. 2.43 ± 0.39, *p* = 0.03); [^18^]DPA-714 did not (2.80 ± 0.69 *vs*. 2.26 ± 0.41). Kinetic modelling with a contralateral reference region identified significantly higher binding potential (BP_ND_) in the core of the LPS injection site with [^18^F]GE-180 but not with [^18^F]DPA-714 *vs*. (R)-[^11^C]PK11195. A cerebellar reference region and novel data-driven input to the SRTM were unable to distinguish differences in tracer BP_ND_.

**Conclusions:**

Second-generation TSPO-PET tracers are able to accurately detect mild-level NI. In this model, [^18^F]GE-180 shows a higher core/contralateral ratio and BP_ND_ when compared to (R)-[^11^C]PK11195, while [^18^F]DPA-714 did not.

**Electronic supplementary material:**

The online version of this article (doi:10.1007/s11307-016-0984-3) contains supplementary material, which is available to authorized users.

## Introduction

Inflammation is known to play a role in the development and progression of a wide variety of neuropathological conditions, including stroke [1], multiple sclerosis [2] and neurodegenerative diseases [3, 4]. Sensitive and spatially accurate imaging of neuroinflammation (NI) *in vivo*, however, has long been a challenge. Microglia, which are resident immune cells of the brain, are in a state of ramification and constantly probing against potential insult or injury, even in the healthy brain [5–7]. Once activated, they begin to alter their morphology, becoming increasingly macrophage-like and releasing pro-inflammatory cytokines such as interleukin-1 (IL-1) and tumour necrosis factor-α (TNF-α). Whether this cytokine cascade exacerbates damage or helps to restore equilibrium conditions varies depending on the nature of the insult and with time [8]. The 18 kDa translocator protein (TSPO), a mitochondrial protein which is primarily located in the membranes of mitochondria [9], is expressed at a low level in the healthy mammalian brain. Although the true role of TSPO in NI remains to be fully understood, an increase in expression of the protein is well established as a correlate with the level of microglial activation, proliferation and/or level of infiltrated macrophages, and to some extent astrogliosis [2, 10–12].

Positron emission tomography (PET) studies of neuroinflammation have been conducted for over 30 years. The original and still most widely used radioligand for TSPO in clinical imaging is the C-11 labelled R-enantiomer of 1-(2-chlorophenyl)-*N*-methyl-*N*-(1-methylpropyl)-3-isoquinoline carboxamide, (R)-[^11^C]PK11195. Despite its application to a wide range of diseases in the clinic, the sensitivity and quantification of this tracer have been hampered by its high level of non-specific binding and low free fraction in plasma in humans, which lead to a poor signal to noise ratio [13]. Additionally, labelling of the ligand with C-11 requires an on-site cyclotron and its radiosynthesis is well-known to be troublesome. In recent years, therefore, efforts have been made to develop and characterise so-called ‘second generation’ TSPO ligands with lower non-specific binding, which have exhibited varying levels of success in clinical and preclinical studies. Unfortunately, and unlike (R)-[^11^C]PK11195, second-generation TSPO ligands are affected by a single-nucleotide polymorphism (rs6971, *Ala147Thr*) in the TSPO gene which results in variable binding affinity in humans [14] but which has never been reported in animals. One such ligand is *N*,*N*-diethyl-2-[4-(2-[^18^F]fluoroethoxy)phenyl]-5,7-dimethyl-pyrazolo[1,5-*a*]pyrimidine-3-acetamide ([^18^F]DPA-714) [15], which was initially evaluated *in vivo* in a rodent model of acute neuroinflammation using α-amino-3-hydroxy-5-methyl-4-isoxazolepropionic acid (AMPA) [16] and then in models of stroke and Herpes encephalitis [17, 18]. Compared to (R)-[^11^C]PK11195, the tracer showed lower non-specific binding and improved bioavailability in brain tissue in the AMPA and stroke models, leading to a better signal to noise ratio than (R)-[^11^C]PK11195; however, this was not the case in the Herpes encephalitis model. Moreover, a more recent preclinical study using [^18^F]DPA-714 in a longitudinal model of HIV-1 in transgenic rats showed no significant group differences in tracer uptake between diseased and wild-type animals [19]. Altogether, these reports support the idea that lower and diffuse NI as observed in neurodegenerative diseases is more variable and difficult to detect [16–18]. Nevertheless, the initial evaluation in humans also suggests that this tracer is a promising TSPO ligand [20] and recently a large clinical study in Alzheimer’s disease (AD) patients showed that neuroinflammation was present in both prodromal and confirmed AD patients [21]. Another second generation tracer, *S*-*N,N*-diethyl-9-[2-[^18^F]fluoroethyl]-5-methoxy 2,3,4,9-tetrahydro-1H-carbazole-4-carboxamide ([^18^F]GE-180), has been used in *in vitro*, *ex vivo* and *in vivo* studies on the detection of microglial activation in acute models of NI [22, 23]. Both studies found an improved signal to noise ratio of [^18^F]GE-180 over (R)- [^11^C]PK11195, but both also used models that induced strong and acute NI (i.e., high dose of lipopolysaccharide (LPS) and stroke) and not the more challenging low/mild NI. Another study in a transgenic AD mouse model, however, did identify increased binding in aged wild-type and AD animals, although this result was semi-quantitative and did not directly compare the behaviour of [^18^F]GE-180 with other tracers [24].

As mentioned above, most of the previous preclinical studies testing second-generation TSPO tracers have primarily used either acute excitotoxic lesion models or models of stroke relevant to the strong levels of NI observed in stroke or brain trauma. In neurodegenerative diseases and models of neurodegeneration, however, the level of TSPO expression is much lower and more widespread than in focal lesions [25–27] and is thus more difficult to detect and quantify. Hence, there is a need to develop an easy-to-perform, robust preclinical model, with low levels of TSPO expression that are clinically relevant to those observed in neurodegeneration. To this end, we performed a comparative evaluation of (R)-[^11^C]PK11195, [^18^F]DPA-714 and [^18^F]GE-180 in rodents injected in the striatum with a low dose (1 μg) of LPS that induces a lower level of neuroinflammation when compared to previously published doses (50 μg [28]), and animals were scanned 3 days post-injection which is after the peak of neuroinflammation induced by LPS between 6 and 12 h post-injection [22, 28].

Kinetic analysis and modelling of preclinical PET data is difficult; as well as being technically challenging, arterial sampling of rodents is terminal, while reference tissue models using the contralateral hemisphere [16, 29] as input are hampered by the fact that the region may not be devoid of specific binding in certain disease models. Moreover, Folkersma et al. [30] suggest that PET data from any disease model where blood brain barrier (BBB) damage is present cannot be quantified using a reference tissue approach with anatomical reference input. Many studies have resorted to semi-quantitation, including use of standardised uptake values (SUVs) [31] and lesion-to-background ratios [32]. There is no consensus over tracer or model-specific quantification approaches, but previous studies involving a unilateral effect have used the cerebellum [33] or a contralateral reference region [22, 23] together with the simplified reference tissue model (SRTM) or MRTM (multilinear reference tissue model) [34]. However, there is, to date, no such evaluation in a model of low-level inflammation where the target *vs*. the contralateral reference region is lower and identification and quantification of the lesion is more challenging.

Indeed, due to the presence of potentially widespread brain inflammation, these anatomically defined reference regions may also contain TSPO in disease. Therefore, data-driven methods have previously been employed in clinical (R)-[^11^C]PK11195 brain PET studies to extract reference tissue kinetics in place of anatomically defined regions (see “Methods” section for more details and [35] for a review). Here, we sought to adapt one such approach [36] for the analysis of rodent PET brain scans in our model of modified LPS injection with the three different TSPO tracers.

Thus, the overall aims of this study were to (*i*) assess the performance of second-generation TSPO tracers [^18^F]GE-180 and [^18^F]DPA-714, directly compared to (R)-[^11^C]PK11195 in a modified LPS model of mild-level neuroinflammation and (*ii*) test various quantification approaches in this model of NI.

## Materials and Methods

### Tracer Synthesis

**Fig. 1.**
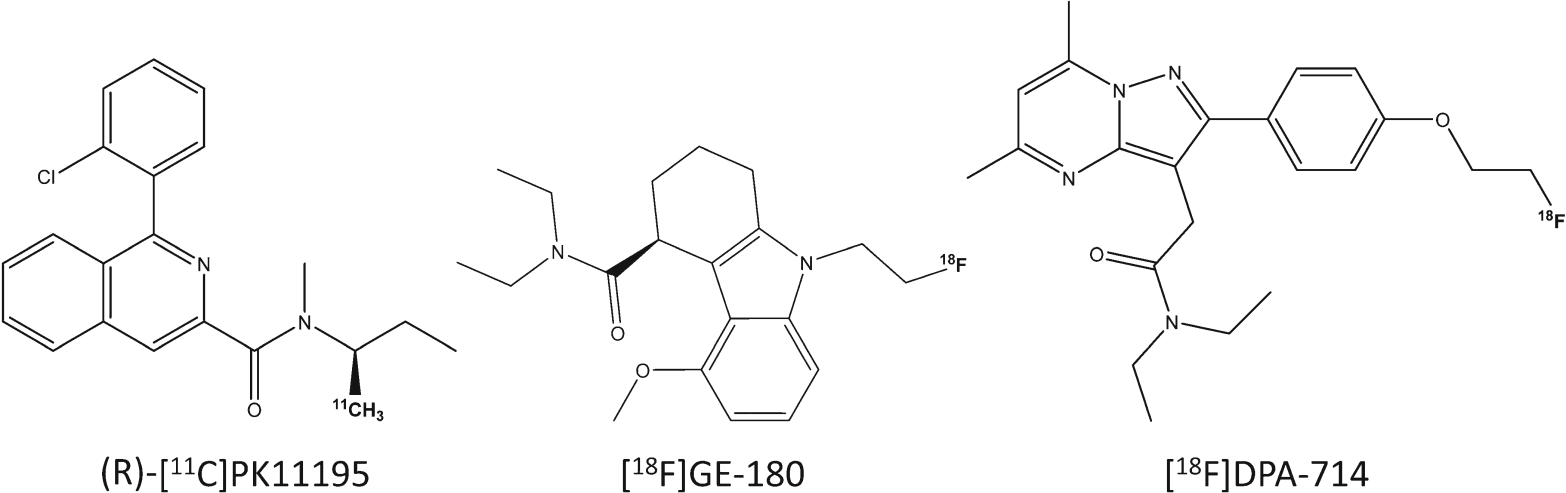
Chemical structures of TSPO PET radiotracers

(R)-[^11^C]PK11195, [^18^F]DPA-714 and [^18^F]GE-180 (Fig. 1) were synthesised as described elsewhere [15, 23, 37–39]. Briefly, (R)-[^11^C]PK11195 was formed via *N*-[^11^C]methylation of an (*R*)*-*demethyl precursor [37, 40]; [^18^F]GE-180 was synthesised on a FASTlab™ platform by nucleophilic fluorination of an *S-*enantiomer mesylate precursor [39, 41] and [^18^F]DPA-714 by nucleophilic aliphatic substitution of a tosylate precursor [15].

### Animals

Thirty-seven adult male Wistar rats (396 ± 52 g, Charles River, Margate, Kent, UK) were used in this study: 22 received an LPS injection, 2 received an AMPA injection, 5 naïve (no stereotactic injection) rats were scanned at baseline as controls with [^18^F]GE-180 (data re-analysed from that previously published [23]), 4 with [^18^F]DPA-714 and 4 with (R)-[^11^C]PK11195. All procedures were carried out in accordance with the Animals (Scientific Procedures) Act 1986, and the project was approved specifically by the UK Home Office. Animals were kept under a 12-h light-dark cycle with free access to food and water.

### LPS and AMPA Administration

For all procedures, animals were anaesthetised by isoflurane inhalation (induction 5 % and thereafter 2–2.5 %) in O_2_/NO_2_ (30 %/70 %). Animals underwent a stereotactic injection of 1 μg lipopolysaccharide (lipopolysaccharide from *Escherichia coli* 055:B5, Sigma, ref. L2880, Lot 102M4017V) or 7.5 nmol (0.5 μl of a 15 mM solution) of AMPA (*n* = 2) in the right striatum via a craniotomy (bregma +0.7 mm, lateral −3.0 mm, depth 5.5 mm from the surface of the brain) using a 2 μl Neuros™ microsyringe (Hamilton, USA) and micropump (injection rate 0.5 μl/min, UltraMicroPump II® and Micro4® Controller, WPI Inc., USA). Animals were maintained normothermic (body temperature 36.5 ± 0.5 °C, mean ± SD) during the surgery through the use of a heating blanket (Homeothermic Blanket Control Unit, Harvard Apparatus Limited®, Edenbridge, Kent, UK).

### Scanning Protocol

**Fig. 2.**
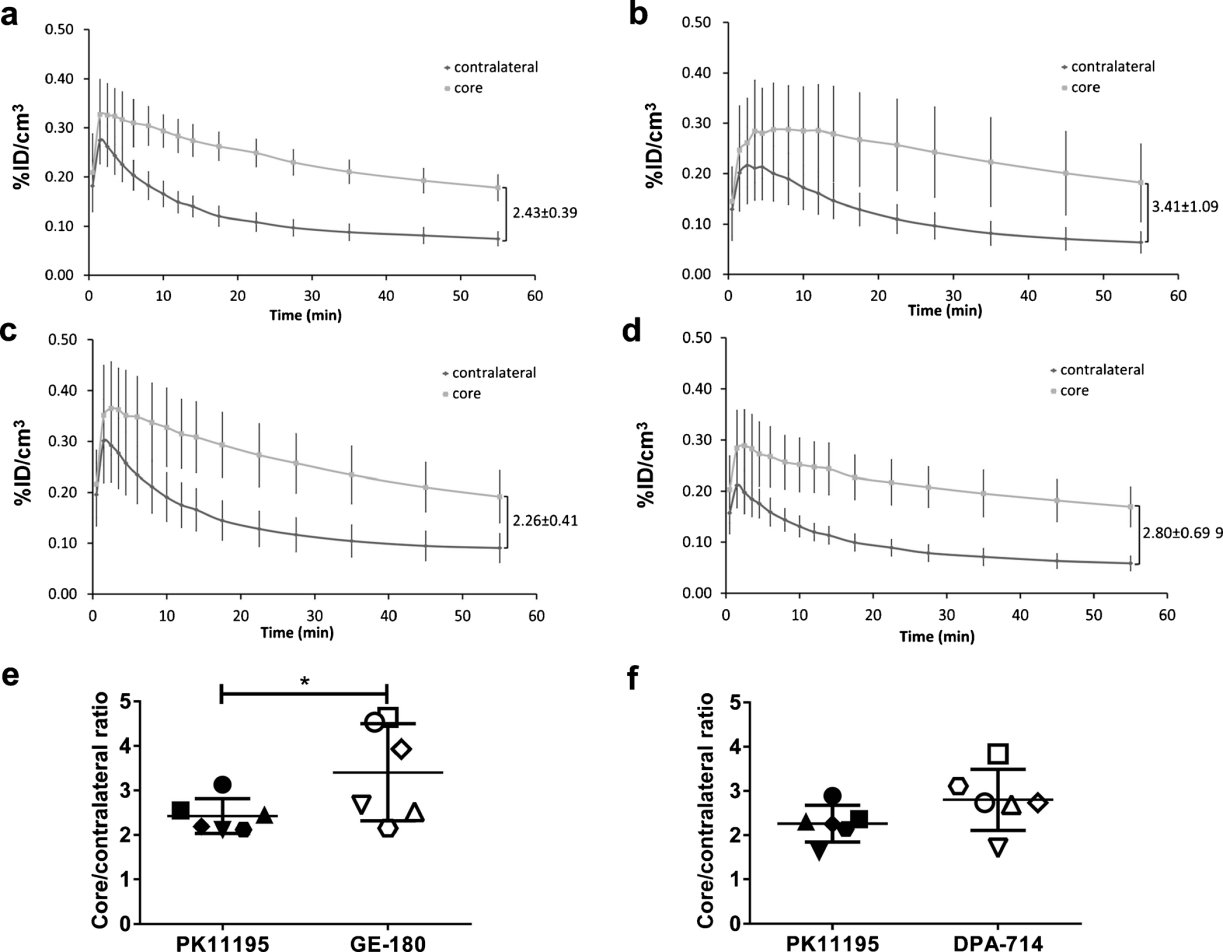
Average core and contralateral tissue time activity curves (TACs) of LPS-injected animals that underwent dual scans with each tracer are shown. TACs are for **a** (R)-[^11^C]PK11195_[_^18^_F]GE-180_, **b** [^18^F]GE-180, **c** (R)-[^11^C]PK11195_[_^18^_F]DPA-714_ and **d** [^18^F]DPA-714 over 60 min duration of scans. Core/contralateral ratios indicated on the figures are calculated over the 40–60 min time frame. Dual scan [^18^F]GE-180 core/contralateral ratios were significantly higher than **e** (R)-[^11^C]PK11195 (*p* = 0.03), while **f** [^18^F]DPA-714 values were not (Wilcoxon signed rank, *p* < 0.05). Individual animals are represented by *different symbols*, which correspond between (R)-[^11^C]PK11195 and the respective F-18 tracer. Data are presented as mean ± SD

**Fig. 3.**
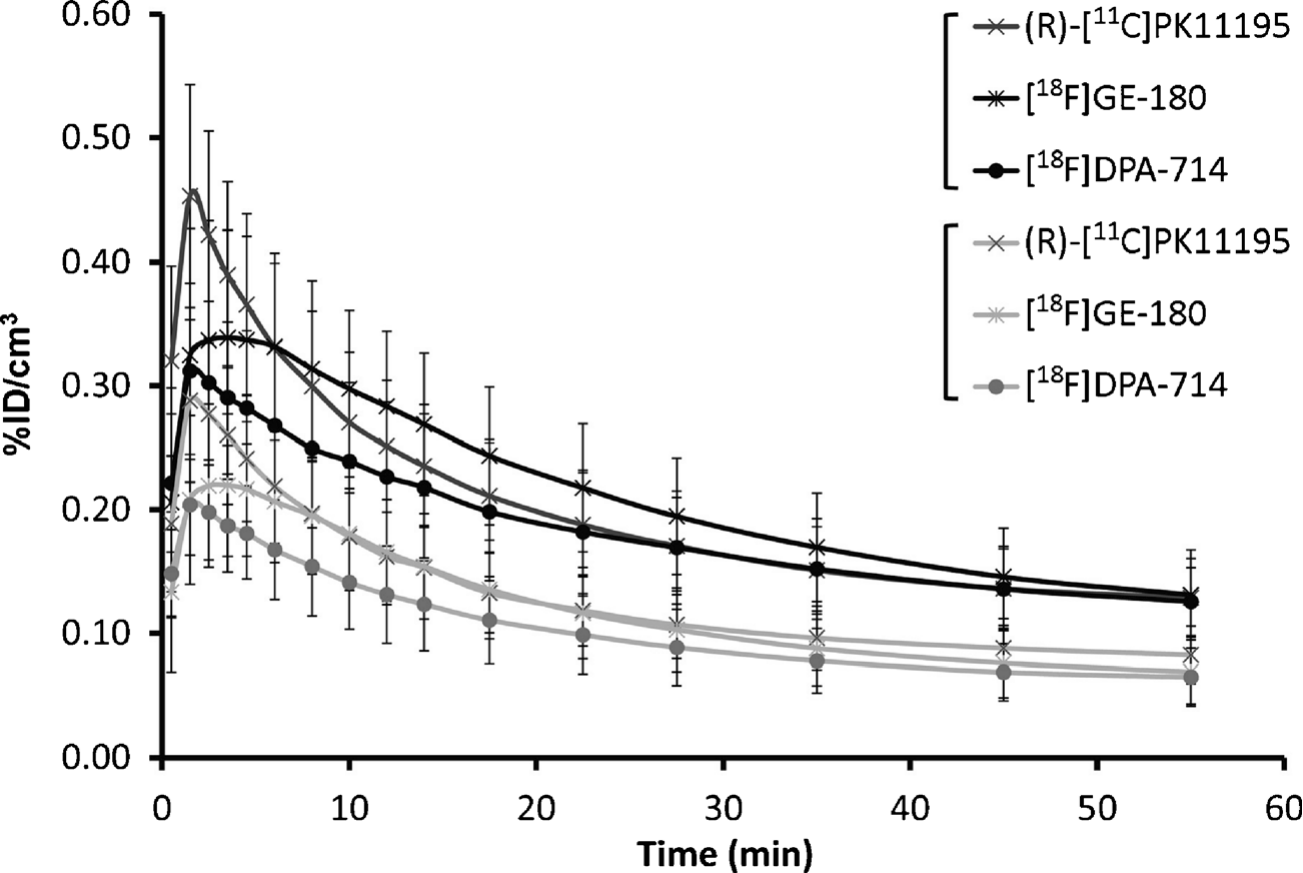
Average TACs for cerebellum (*black*) and contralateral (*grey*) reference regions for (R)-[^11^C]PK11195, [^18^F]GE-180 and [^18^F]DPA-714 in LPS animals. At 40–60 min, the cerebellar uptake (%ID/cm^3^) was significantly higher for all three tracers (*p* = 0.001) than the contralateral uptake

**Fig. 4.**
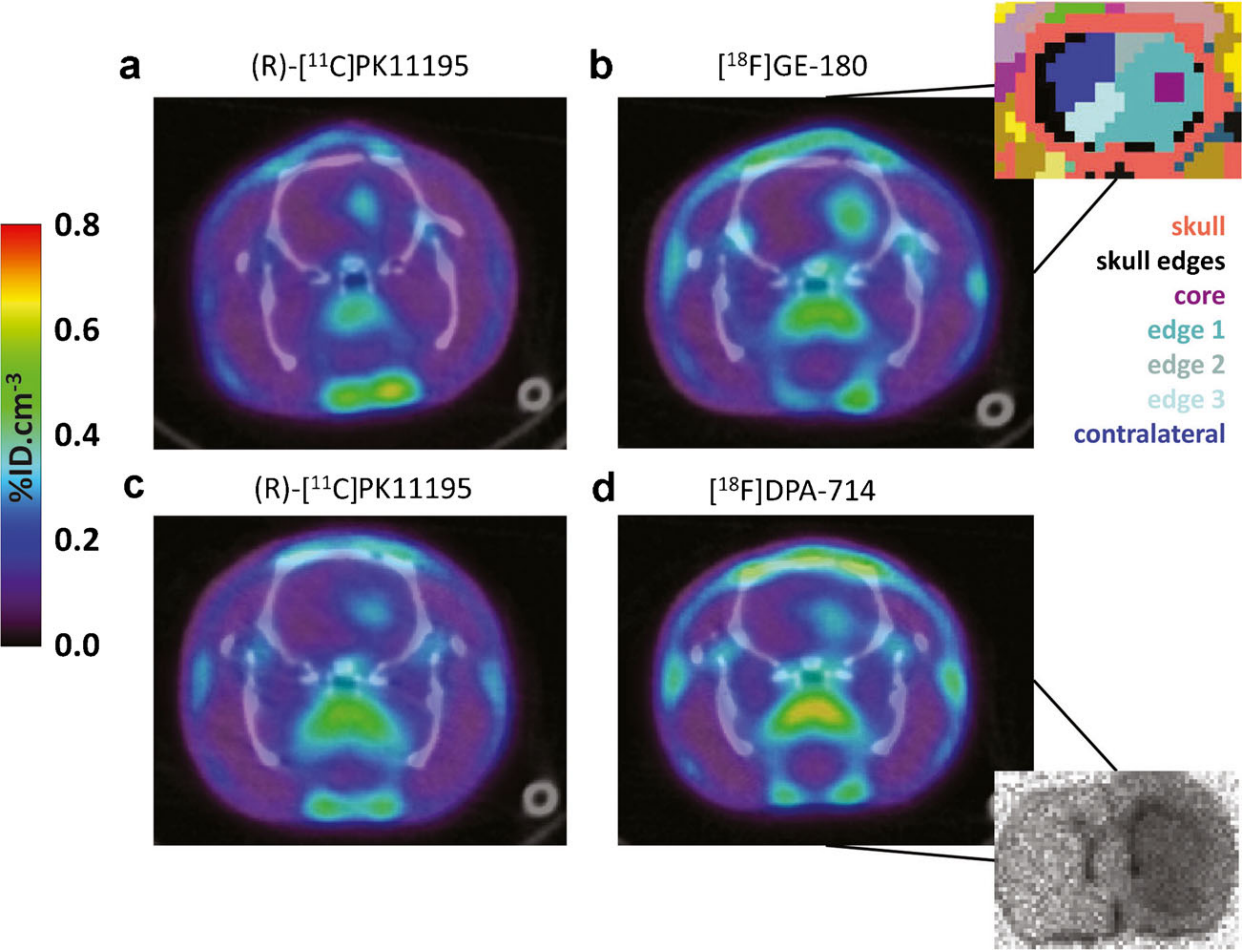
Co-registered (sum 40–60 min) PET/CT images of representative animals dual-scanned with **a** and **c** (R)-[^11^C]PK11195 followed by either **b** [^18^F]GE-180 (top) or **d** [^18^F]DPA-714 (*bottom*). *Inset top*: example of regions defined by automatic segmentation. *Inset bottom*: [^18^F]DPA-714 autoradiographic image showing increased specific uptake in the right striatum

Supplementary Fig. 1 presents a summary of study design; full details of the tracer injected doses, specific activities and purities can be found in Supplementary Table 1. Animals were split into four groups: those scanned sequentially with (R)-[^11^C]PK11195 then [^18^F]GE-180 (group 1, *n* = 6) or (R)-[^11^C]PK11195 then [^18^F]DPA-714 (group 2, *n* = 6) and those scanned with only [^18^F]GE-180 (group 3, *n* = 5) or [^18^F]DPA-714 (group 4, *n* = 5). In groups 1 and 2, animals were scanned on the same day with (R)-[^11^C]PK11195 in the morning and an F-18 labelled tracer in the afternoon. Supplementary Fig. 2 shows a correlation plot between specific activity and percentage injected dose of tracer in the inflammatory core at 40–60 min. Although the range of specific activities was quite broad, especially for (R)-[^11^C]PK11195, this did not significantly affect brain uptake (Figs. 2, 3, 4 and Supplementary Fig. 2).

All animals were anaesthetised by isoflurane inhalation (induction 5 % and thereafter 2–2.5 %) in O_2_/NO_2_ (30 %/70 %) at 3 days post-LPS injection. All tracers were injected intravenously as a bolus through a tail vein. They were then scanned on a Siemens Inveon® small animal PET-CT as described previously [23]. The following data acquisition protocol was used: a CT scan was performed immediately prior to the PET scan for each animal to acquire attenuation correction factors. The time coincidence window was set to 3.432 ns and levels of energy discrimination to 350 and 650 keV. List mode data from emission scans were histogrammed into 16 dynamic frames (5 × 1 min, 5 × 2 min, 3 × 5 min and 3 × 10 min) and emission sinograms were normalised, corrected for attenuation, scattering and radioactivity decay and reconstructed using OSEM3D (16 subsets, 4 iterations) into 128 × 128 × 159 images with 0.776 × 0.776 × 0.796 mm^3^ voxel size. Respiration and temperature were monitored throughout the scans using a pressure-sensitive pad and rectal probe (BioVet, M2M Imaging Corp, USA). Body temperature was maintained through the use of a heating and fan module controlled by the rectal probe via the interface of the BioVet system.

At the end of the PET scan, rats were rapidly decapitated and brains removed and frozen immediately in isopentane in dry ice. Brains were stored at −80 °C before being cut with a cryomicrotome into adjacent 20 μm coronal slices. Sections were then stored again at −80 °C until immunohistochemistry and autoradiography were performed.

### Image Analysis

Images were segmented automatically using local means analysis (LMA) in the BrainVisa/Anatomist framework (http://brainvisa.info, [42–44]). Automatic segmentation was preferred to manually drawn regions of interest (ROIs), which can introduce a user-dependent bias. In short, the LMA algorithm first extracts the whole body of the rodent from the background before identifying voxels in the core of organs based on the notion that their variation in PET signal should be lower than those voxels at the organ borders, which are more subject to variations driven by physiological movement and spillover from neighbouring organs/vessels. Neighbouring voxels are then identified based on the similarity of their kinetics to these organ ‘cores.’ Ultimately, the implementation in BrainVisa segments the whole body image into 200 regions based on the similarity of kinetics of the voxels contained within them. Within the brain (delineated using the CT images), segmented ROIs were then manually selected and labelled under the following scheme: (1) core of the LPS lesion (covering the right striatum and/or the region with the highest uptake), (2) edge 1 (ROI around the core with the second highest uptake of tracer), (3) edge 2 (ROI with the third highest uptake), (4) a contralateral region (ROI with the lowest uptake, used as a reference region in kinetic analysis), (5) cerebellum and (6) skull edges (voxels on the edges of the skull taken to be noise and/or spillover from regions outside the brain, not included in the data analysis) (see top-right corner inset Fig. 4). Where applicable, a region of fourth highest uptake (edge 3) was also included in ROI segmentation. In the control animals, regions of high and low brain uptakes, as well as cerebellum and skull edges, were defined. ROIs’ volumes were similar for all tracers (core 86 ± 20 mm^3^, edge 1 202 ± 3 mm^3^, edge 2 498 ± 114 mm^3^, edge 3 268 ± 31 mm^3^ and contralateral 217 ± 16 mm^3^, with a maximum variation in ROIs’ volumes across tracers of 23 %).

The previous work published by our group has presented results with partial volume correction (PVC). Although in our previous study with high amplitude response in TSPO expression, group differences were not affected by PVC being applied or not [23], for low level NI models PVC might actually reduce the signal to background ratio and therefore damp the PET signal. This is due to PVC inherently amplifying noisy, high-frequency signals to reduce spill-over effects. Therefore, in our model of low/mild NI, applying PVC might be somewhat counter-productive. Having analysed results with and without PVC, here we selected to present raw (uncorrected) data, which showed consistent group differences between tracers.

### Data-Driven Method for the Extraction of Reference Tissue Kinetics

The method by Yaqub et al. [36] is a successor to that by Turkheimer et al. [45], using a pre-defined brain mask to segment all masked voxel PET time activity curves (TACs) into four classes (grey matter with specific binding, grey matter without specific binding, white matter and blood). Subsequently, in dynamic PET data, the reference tissue is selected as the tissue class with the kinetic most similar to that of grey matter without specific binding. This method requires segmented MR data for the generation of a (camera-specific) population database. Since the spatial resolution of the Inveon® small animal PET-CT camera is between 1 and 2 mm, it is not possible to resolve distinct grey and white matter kinetics in the rat brain. Therefore, we replaced the MR-guided tissue segmentation [36, 45] with an automated kinetic data segmentation approach developed for preclinical PET data (LMA) [42–44]. The LMA method, implemented in BrainVisa, produced three classes of sufficient size (over 10 % of total brain voxels) in the rat brain and with voxels which were located in spatial proximity to each other. We additionally attempted to define more classes (as used in clinical supervised clustering [36, 45]); however, no more than three kinetically distinct classes were visible in the stroke rodent brain (identifiable by TAC shape), suggesting that this was the optimum number for class definition. This was consistent for (R)-[^11^C]PK11195, [^18^F]GE-180 and [^18^F]DPA-714.

In our implementation, a population of adult male Wistar rats in a model of focal cerebral ischaemia were used for class definition. Six animals underwent transient right middle cerebral artery occlusion (MCAO) for 60 min (data published previously and re-analysed [23]) and were dual scanned with (R)-[^11^C]PK11195 and [^18^F]GE-180 5 or 6 days later. In addition, four animals with MCAO were scanned with (R)-[^11^C]PK11195 and [^18^F]DPA-714 (data re-analysed from [17]). All data were reconstructed and pre-processed as described above. Brain masks [36] were extracted from the individual CT images for each animal. The classes were defined within the brain as ‘activated tissue’ represented by the core of the infarct; ‘normal tissue’ from the contralateral ROI; and ‘tissue with intermediate binding’, represented by cerebral tissue located between the core and contralateral tissue and in the cerebellum. As described above, these regions were defined using the BrainVISA LMA segmentation, for each animal, and TACs were normalised (by subtracting from each frame its mean and dividing it by its standard deviation, SD, to create a unit input) and averaged to produce a population database. The algorithm was then implemented on the dynamic images using the Matlab code, SuperPK Software (Imperial Innovations, Imperial College London, UK [45, 46], developed for clinical studies and adapted here for preclinical class definitions), from the LPS animals to produce cluster maps for normal, intermediate and activated tissue, as well as a reference kinetic, based on the class of voxel most kinetically similar to normal tissue identified in the training data set, for input to the SRTM.

### PET Data Modelling

Kinetic analysis of data was performed using the SRTM [47] in the PMOD software package (version 3.6, PMOD Technologies Ltd.) for ROIs (1) to (5). The model assumes a 1-tissue compartment arrangement in the target region (lesion) and the reference region. Input functions were derived using the contralateral hemisphere ROI (region 4), cerebellum (region 5) and the supervised clustering approach. *R*_1_ (ratio of tracer delivery in the target ROI *vs*. that in all respective reference regions) and BP_ND_ were calculated for all ROIs.

### Immunohistochemistry

Brain sections, including the striatum, from five LPS animals which were also scanned *in vivo* were stained for CD11b and glial fibrillary acidic protein (GFAP) to confirm the presence of microglia and astrocytes respectively. Staining for neuronal nuclei was also completed using an anti-NeuN antibody. Sections from four animals were also stained to investigate BBB integrity: visualising tight junctions with claudin-5 and large protein diffusion with IgG.

For all procedures described below, phosphate buffered saline (PBS) at 100 mM was used. Rat brain sections were snap frozen in isopentane and stored at −80 °C. Sections were then post-fixed in paraformaldehyde (4 % in PBS) for 10 min and washed (3 × 5 min) in PBS. Sections were incubated in 1 mg/ml sodium borohydride in PBS (3 × 5 min) to reduce auto-fluorescence and washed again in PBS (3 × 5 min). They were then permeabilised with 2 h of incubation in 0.1 % Triton X-100 containing 3 % normal donkey serum in PBS to block non-specific binding. Without further washing, sections were incubated overnight at 4 °C with primary antibodies in 3 % normal donkey serum/0.1 % Triton X-100 in PBS. Double immunohistochemical staining was performed against GFAP with rabbit anti-cow GFAP (Dako, 1:400) and CD11b (OX42) with mouse anti-rat CD11b (Serotec, 1:1000). Adjacent sections from the same animals were stained with mouse anti-mouse NeuN (Chemicon, 1:1000). Sections were then washed (3 × 10 min) in PBS/Triton and incubated for 2 h at room temperature with secondary antibodies (AlexaFluor® 488 nm donkey anti-rabbit for GFAP, AlexaFluor® 594 nm donkey anti-mouse for CD11b, or AlexaFluor® 594 nm donkey anti-mouse for NeuN (Molecular Probes, Invitrogen)), all 1:500 in 3 % normal donkey serum/0.1 % Triton X-100 in PBS and then washed again (3 × 10 min) in PBS.

Following the same protocol, brain sections from two animals injected with LPS from the *in vivo* study and two animals injected with AMPA (positive control) were incubated with monoclonal rabbit anti-claudin-5 (Sigma-Aldrich, 1:100) primary antibody and AlexaFluor® 488 nm donkey anti-rabbit. To stain for endogenous rat IgG infiltration in the brain parenchyma, adjacent sections were incubated with AlexaFluor® 594 nm donkey anti-rat. AMPA-injected animals were of the same strain and comparative age/weight to LPS animals. Supplementary Table 2 provides a summary of primary and secondary antibodies used during immunohistochemistry.

All sections were mounted with a Prolong® Gold Antifade kit (Molecular Probes, Invitrogen). Images were collected on an Olympus BX51 upright microscope using 4×/0.13, 10×/0.30 or 40×/0.50 UPlanFLN objectives and captured using a Q-Capture Retiga 6000 camera through Q-Capture Pro Software (Molecular Devices). Specific band pass filter sets were used to prevent bleed through from one channel to the next. Images were processed and analysed using ImageJ (http://rsb.info.nih.gov/ij).

### Autoradiography

In order to confirm the presence of specific TSPO binding, [^18^F]DPA-714 autoradiography was performed with 20-μm brain sections from five LPS animals (adjacent to those used for immunohistochemistry). Sections were first incubated for 5 min in cold Tris buffer (Trizma crystals (Sigma, UK), 50 mM adjusted to pH 7.4 at 4 °C with 120 mM NaCl) and then incubated for 1 h at room temperature with either [^18^F]DPA-714 (total binding) (98.7 GBq/μmol; 5 nM) or [^18^F]DPA-714 with excess PK11195 (5 μM) to assess non-specific binding. Sections were rinsed twice for 2 min with cold buffer and then once quickly with cold distilled water, before being dried and exposed overnight to a Phosphor-Imager screen (Fujifilm BAS-1800II, Raytek Scientific Ltd., Sheffield, UK). Autoradiographs were visualised and analysed using AIDA software (Raytest GmbH, Germany). ROIs were drawn manually on the lesion and the contralateral area, and binding is expressed as the intensity of photostimulated luminescence (PSL) per pixel.

### Statistical Analysis

All data are expressed as mean ± SD. Paired Wilcoxon signed rank tests were used to compare (R)-[^11^C]PK11195 and the corresponding F-18 tracer uptake or BP_ND_ values obtained in the same animal (dual tracer scans). Non-paired (R)-[^11^C]PK11195, [^18^F]GE-180 and [^18^F]DPA-714 results were compared using the Mann-Whitney *U* test. Non-paired comparisons of tracers were performed as (R)-[^11^C]PK11195_[_^18^_F]GE-180_ (group 1) *vs*. [^18^F]DPA-714_only_ (group 4) and (R)-[^11^C]PK11195_[_^18^_F]DPA-714_ (group 2) *vs*. [^18^F]GE-180_only_ (group 3) (see Supplementary Table 1). Comparisons between all animals scanned with [^18^F]GE-180 and [^18^F]DPA-714 (*n* = 11 *vs*. *n* = 11) were also performed. Statistics were performed in GraphPad Prism for Windows (v6.04, GraphPad Software, Inc., San Diego, CA, USA).

## Results

### PET Imaging

All PET images in LPS animals (groups 1–4) showed localised increase in tracer uptake (%ID/cm^3^) in the core ROI (Figs. 2 and 4, Table 1). Furthermore, the uptake in edges 1, 2 and 3 was lower compared to the core and not statistically different than the contralateral side, indicating a gradual decrease in neuroinflammation with distance from the injection site.

**Table 1:** Regional uptakes expressed in %ID/cm^3^ and core/contralateral ratio for each group (40–60 min sum image). Data expressed as mean ± SD

Group	Tracer	Core	Contralateral	Core/contralateral ratio	Cerebellum	Edge 1	Edge 2	Edge 3
1	(R)-[^11^C]PK11195_([_^18^_F]GE-180)_	0.19 ± 0.03	0.08 ± 0.02	2.43 ± 0.39	0.12 ± 0.02	0.14 ± 0.03	0.11 ± 0.02	0.08 ± 0.02
	[^18^F]GE-180	0.24 ± 0.08	0.08 ± 0.03	3.41 ± 1.09*	0.13 ± 0.04	0.16 ± 0.04	0.11 ± 0.03	0.08 ± 0.03
2	(R)-[^11^C]PK11195(_[_^18^_F]DPA-714)_	0.20 ± 0.05	0.09 ± 0.03	2.26 ± 0.41	0.14 ± 0.04	0.16 ± 0.05	0.13 ± 0.05	0.11 ± 0.04
	[^18^F]DPA-714	0.19 ± 0.03	0.07 ± 0.03	2.80 ± 0.69	0.14 ± 0.03	0.14 ± 0.03	0.12 ± 0.05	0.09 ± 0.05
3	[^18^F]GE-180_only_	0.19 ± 0.08	0.07 ± 0.02	2.76 ± 0.48	0.15 ± 0.04	0.15 ± 0.05	0.10 ± 0.03	0.09 ± 0.03
4	[^18^F]DPA-714_only_	0.18 ± 0.04	0.06 ± 0.01	2.92 ± 0.43	0.12 ± 0.02	0.13 ± 0.03	0.09 ± 0.02	0.09 ± 0.06

*Asterisk* indicates BP_ND_ values significantly different from (R)-[^11^C]PK11195 (paired Wilcoxon signed rank test, *p* < 0.05)

### Non-Paired Scans

For non-paired scans, there was no significant difference in 40–60 min core *or* contralateral uptakes between tracers. There were no significant differences between the core/contralateral uptake ratios at 40–60 min between (R)-[^11^C]PK11195 and either F-18 tracer when considering non-paired groups of animals: (R)-[^11^C]PK11195_[_^18^_F]DPA-714_ (2.26 ± 0.41, *n* = 6) *vs*. [^18^F]GE-180_only_ (2.76 ± 0.48, *n* = 5) or (R)-[^11^C]PK11195_[_^18^_F]GE-180_ (2.43 ± 0.39, *n* = 6) *vs*. [^18^F]DPA-714_only_ (2.92 ± 0.43, *n* = 5). The unpaired comparison of animals scanned with [^18^F]GE-180 (*n* = 11) and animals scanned with [^18^F]DPA-714 (*n* = 11) showed no significant difference in core/contralateral uptake at 40–60 min (*p* = 0.47) or the core/contralateral ratio (Table 1). Additionally, there were no significant differences between animals scanned with [^18^F]GE-180_only_ (*n* = 5) and [^18^F]GE-180 (*n* = 6), nor with [^18^F]DPA-714_only_ (*n* = 5) and [^18^F]DPA-714 (*n* = 6) (Table 1). There was also no significant difference between the contralateral ROI and the edges 1, 2, 3 and cerebellum ROIs (Table 1).

For comparison with the control (naïve) animals, *all* the LPS-injected animals were pooled for each tracer. Average contralateral uptakes in all LPS animals at 40–60 min (0.09 ± 0.02 %, 0.07 ± 0.03 % and 0.07 ± 0.02 % for (R)-[^11^C]PK11195 ((R)-[^11^C]PK11195_[_^18^_F]GE-180_ + (R)-[^11^C]PK11195_[_^18^_F]DPA-714_, *n* = 12), [^18^F]GE-180 (+[^18^F]GE-180_only_ *, n* = 11) and [^18^F]DPA-714 (+[^18^F]DPA-714_only_, *n* = 11) respectively) were not significantly different from the low uptake regions in control animals (0.09 ± 0.01 % (*n* = 4), 0.08 ± 0.01 % (*n* = 5) and 0.05 ± 0.01 % (*n* = 4) for (R)-[^11^C]PK11195, [^18^F]GE-180 and [^18^F]DPA-714 respectively), supporting the use of the contralateral ROI as reference region.

### Dual Scans

For dual scans, there was no significant difference between all the ROIs studied for all tracers. However, core/contralateral ratios were significantly higher (+40 ± 36 %) for [^18^F]GE-180 than (R)-[^11^C]PK11195 (3.41 ± 1.09 *vs*. 2.43 ± 0.39, *p* = 0.03), while [^18^F]DPA-714 values were not (2.80 ± 0.69 *vs*. 2.26 ± 0.41, *p* = 0.09, +25 ± 25 %; Table 1 and Fig. 2).

### Kinetic Analysis

Previous work [17, 23] has shown that only paired scans can truly account for inter-individual variability; thus, for the kinetic analysis, only dual scan data were considered.

### SRTM with Contralateral Reference Input

Table 2 shows BP_ND_ results for dual-scanned animals. Average values calculated with a contralateral reference input for animals scanned with (R)-[^11^C]PK11195 then [^18^F]GE-180 were 1.25 ± 0.29 and 1.94 ± 0.75 respectively (+57 ± 57 %, *p* = 0.03), while (R)-[^11^C]PK11195 *vs*. [^18^F]DPA-714 dual scan BP_ND_ values were 1.11 ± 0.31 and 1.58 ± 0.54, respectively (+43 ± 34 %, *p* = 0.06).

**Table 2:** BPND values from the simplified reference tissue model in the core ROI with different reference tissues: dual scans. Data expressed as mean ± SD

Reference region	(R)-[^11^C]PK11195_[_^18^_F]GE-180_	[^18^F]GE-180	(R)-[^11^C]PK11195_[_^18^_F]DPA-714_	[^18^F]DPA-714
Contralateral	1.25 ± 0.29	1.94 ± 0.75*	1.11 ± 0.31	1.58 ± 0.54
Cerebellum	0.60 ± 0.26	0.84 ± 0.46	0.46 ± 0.17	0.43 ± 0.26
Supervised clustering	0.83 ± 0.22	0.64 ± 0.42	0.75 ± 0.20	0.49 ± 0.20*

*Asterisk* indicates BP_ND_ values significantly different from (R)-[^11^C]PK11195 (paired Wilcoxon signed rank test, *p* < 0.05)

### SRTM with Cerebellar Reference Input

Overall, using a cerebellar reference input returned lower BP_ND_ values than using a contralateral reference input (*p* = 0.001 for all tracers) and BP_ND_ results were not significantly different between tracers (0.60 ± 0.26 *vs*. 0.84 ± 0.46 for (R)-[^11^C]PK11195 *vs*. [^18^F]GE-180; 0.46 ± 0.17 *vs*. 0.43 ± 0.26 for (R)-[^11^C]PK11195 *vs*. [^18^F]DPA-714, Table 2).

### SRTM with Data-Driven Clustering Reference Input

BP_ND_ values with a data-driven reference input function were overall lower than those from a contralateral reference region (*p* = 0.001 for (R)-[^11^C]PK11195 and [^18^F]DPA-714, and *p* = 0.002 for [^18^F]GE-180), but were highly correlated (Spearman’s correlation coefficient *ρ* = 0.83 and 0.90 respectively for dual scans). For dual-scanned animals, (R)-[^11^C]PK11195 and [^18^F]GE-180 values were 0.83 ± 0.22 and 0.63 ± 0.43 respectively (*p* = 0.31, n.s.), while (R)-[^11^C]PK11195 and [^18^F]DPA-714 BP_ND_ values were 0.75 ± 0.20 and 0.49 ± 0.20 respectively (*p* = 0.03, Table 2).

### Autoradiography

Autoradiographs confirmed the presence of specific [^18^F]DPA-714 binding in the ipsilateral striatum compared to the contralateral striatum. Quantification of the autoradiography revealed a 5.62 ± 0.72-fold increase in specific binding in the ipsilateral striatum when compared to the contralateral striatum (Fig. 4 (insert bottom right) and Supplementary Fig. 3).

### Immunohistochemistry

**Fig. 5.**
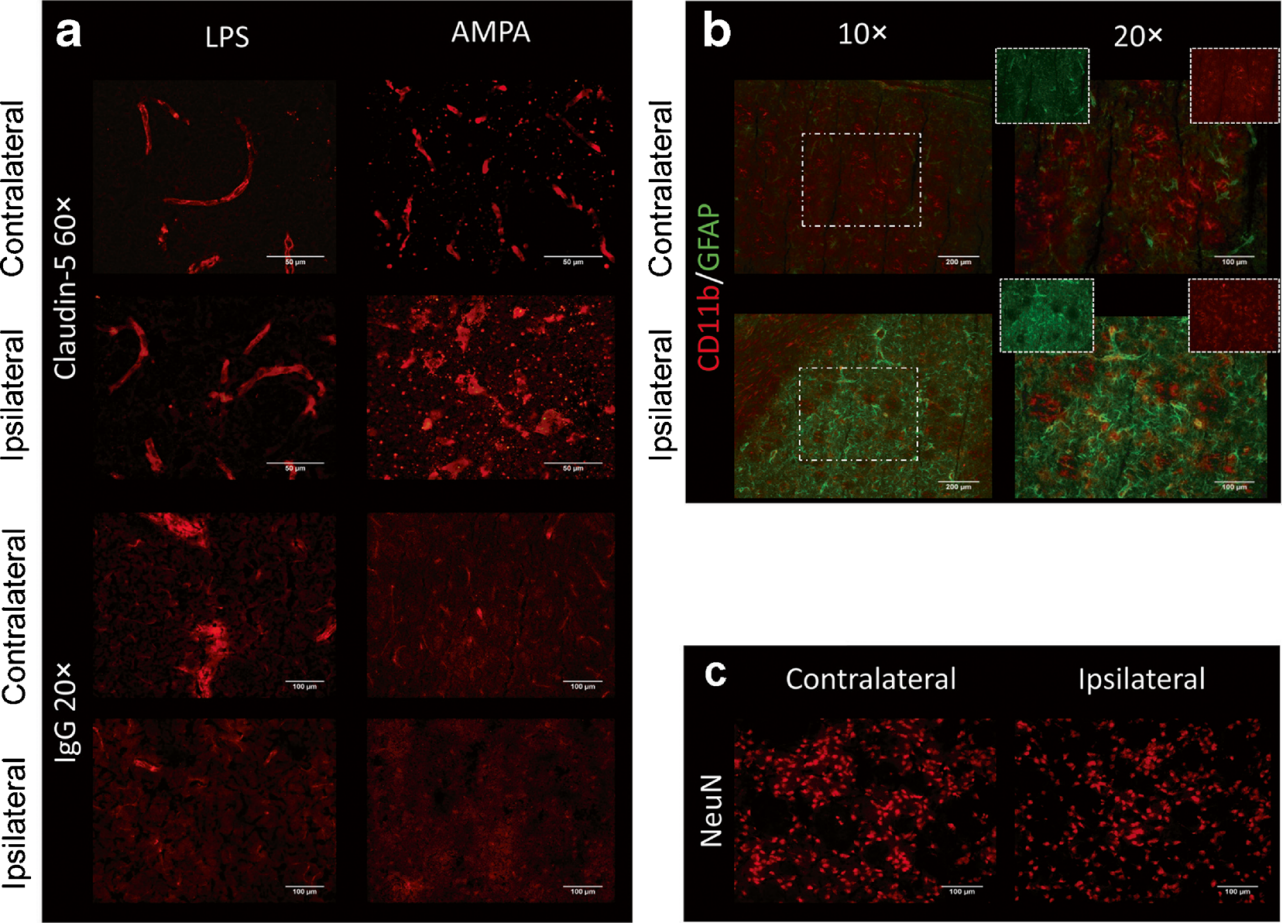
**a** Claudin-5 (×60 magnification) and IgG (×20 magnification) staining in ipsilateral and contralateral striatum for LPS and positive control AMPA-challenged animals. Claudin-5 staining revealed no differences between contralateral and ipsilateral sides in the LPS-challenged animals, while for AMPA-challenged animals, there was visible damage to vessels in the ipsilateral side and some disruption in the contralateral hemisphere. IgG appears normal in ipsilateral and contralateral sides of LPS animals but shows signs of leakage in the ipsilateral side of AMPA animals. **b** Staining for microglia (CD11b, *red*) and astrocytes (GFAP, *green*), merged at ×10 and ×20 (*white rectangles*) magnification; **c** NeuN staining at ×20 magnification in core and contralateral striatum

Anti-rat IgG immunohistochemistry will label rat IgG in blood vessels; as a large protein, IgG should not diffuse into the brain if the BBB is intact. Our staining indicated that rat IgG were still localised in blood vessels in the LPS model, hence supporting presence of an intact BBB, whereas in the AMPA model the staining of blood vessels was lost and was diffuse in the brain parenchyma, indicating BBB leakage. This was confirmed by immunohistochemistry of the tight-junction protein claudin-5, as LPS animals showed similar intact vessels in the ipsilateral and contralateral area, while in AMPA-challenged animals, the vessels were clearly damaged (Fig. 5a) in the ipsilateral side, indicating a compromised BBB.

Activated microglial cells (CD11b) were observed in large numbers in the core of the inflamed area compared to the contralateral hemisphere, as were astrocytes (GFAP) (Fig. 5b). NeuN stained nuclei showed no obvious difference between contralateral and ipsilateral striatum, supporting that there was no neuronal loss induced by the LPS injection (Fig. 5c).

## Discussion

### PET Imaging and *Ex Vivo* Observations

Although results from rodents are not directly transferable to humans due to inter-species differences, preclinical studies are still valuable for assessment and characterisation of tracer behaviour, especially where models can be developed to mimic clinically relevant levels of inflammation. The primary aim of this study was to quantitatively compare the recently developed second-generation TSPO radioligand [^18^F]GE-180 with the more well-established [^18^F]DPA-714 and the original (R)-[^11^C]PK11195 in a rat model of low/mild focal inflammation. Recently published work has shown that both F-18 labelled tracers have an improved SNR over (R)-[^11^C]PK11195 in models of acute inflammation [17, 22, 23, 28], but to date, there is neither reported investigation comparing these new tracers nor looking at their performance in a model producing a lower level inflammation (∼2-fold increase in TSPO PET signal), which is more similar to the levels observed in neurodegenerative diseases than stroke [17, 23], excitotoxic lesions [16, 48] or high doses of LPS [28, 34] (∼4–6-fold increase in TSPO PET signal). The second aim of this study was to assess the kinetic quantitation methodology via use of the SRTM with different reference input functions. Non-terminal preclinical kinetic modelling has so far been limited to semi-quantitation (SUV) or use of an anatomical reference region in unilateral models. In order to evaluate methods that could be used in neurodegenerative diseases, such as AD transgenic models, which display low levels of neuroinflammation and where a contralateral reference tissue does not exist, we have compared here the BP_ND_ values obtained with the SRTM and three different reference input functions: (1) a contralateral region, (2) a cerebellar region, (3) a reference kinetic derived from a data-driven supervised clustering approach.

Previous preclinical studies using an LPS model have typically used larger doses of the endotoxin, inducing high-level inflammation [22, 28]. Moreover, previous publications did not compare (R)-[^11^C]PK11195 with second-generation TSPO tracers [28, 34] in the more challenging setting of low/mild neuroinflammation and animals were not dual scanned with the tracers [22]. Finally, the comparison between (R)-[^11^C]PK11195 and [^18^F]GE-180 performed by Dickens et al. only used the contralateral reference region as input for modelling.

Our study showed that [^18^F]GE-180 uptake and BP_ND_ were significantly higher than those of (R)-[^11^C]PK11195, while [^18^F]DPA-714 were not. Figure 2e–f shows the core/contralateral uptake ratios for dual-scanned animals; it is inferable from Fig. 2e that the significant difference seen between [^18^F]GE-180 and (R)-[^11^C]PK11195 may be driven by three particular animals of the six scanned with both (R)-[^11^C]PK11195 and [^18^F]GE-180. These animals are also likely to drive the increased variability observed with [^18^F]GE-180 over the other tracers. We also performed a comparison in ‘non-paired’ animals individually scanned with different tracers ((R)-[^11^C]PK11195_[_^18^_F]DPA-714_ *vs*. [^18^F]GE-180, (R)-[^11^C]PK11195_[_^18^_F]GE-180_ *vs*. [^18^F]DPA-714, Table 1). Interestingly, core/contralateral uptake ratios between 40 and 60 min post-injection for *unpaired* groups were *not* significantly higher for either [^18^F]GE-180 or [^18^F]DPA-714 when compared to (R)-[^11^C]PK11195, or compared to one another. Altogether, the lack of differences between [^18^F]DPA-714 and [^18^F]GE-180 suggests that both tracers provide similar readouts of neuroinflammation.

These observations also highlight (1) that the differences in sensitivity (if any) between the three tracers used in this study are rather small (2) that, as hypothesised, the inherent variability within an experiment or populations makes quantification more challenging when measuring low/mild level of neuroinflammation and (3) further supports the importance of dual scanning in the same subject to truly compare the performance of tracers both in preclinical and clinical settings.

*Ex vivo* experiments were performed in order to confirm the *in vivo* observations. Firstly, immunohistochemistry was performed with CD11b to assess microglial presence in the core of the LPS injection site, GFAP staining for astrocytic activation, NeuN to evaluate neuronal loss and claudin-5 and IgG to assess BBB disruption. Results supported the PET findings; both activated microglia and astrocytes were significantly present in the right striatum compared to the left (contralateral) side. Autoradiography also confirmed the presence of TSPO specific binding in the ipsilateral striatum while the NeuN staining showed no neuronal loss in the right striatum. Finally, claudin-5 staining showed no BBB disruption compared to a positive control AMPA model (Fig. 5a).

Although this study was designed to directly compare the three tracers *in vivo*, comparison of [^18^F]GE-180 *vs*. [^18^F]DPA-714 in the same animal was not practically feasible due to the rapid progression of TSPO expression over 24 h as induced by LPS injection, requiring the scans to be conducted within hours, and the constraints imposed by the half-life of F-18 and the tracer production. Similarly, considering the difference in tracer pharmacokinetics, the comparison of these two tracers by *in vitro* autoradiography would not have yielded relevant information regarding their *in vivo* behaviour as PET tracers, hence our comparison of unpaired scans for these two tracers.

### PET Data Modelling

In the case of a pathologically damaged BBB, the tracer gains access to the brain parenchyma; thus the extent of binding to TSPO is affected non-trivially and should be accounted for in kinetic modelling. Here, *ex vivo* investigation of the BBB disruption in LPS-challenged animals *vs*. positive control AMPA-challenged animals showed no significant BBB disruption in the inflamed area with this low dose of LPS when compared to the AMPA-treated animals (Fig. 5a). Additionally, *R*_1_ values calculated using the contralateral reference region were close to unity in the core of the inflamed area for all animals, suggesting that tracer diffusion to the contralateral (reference) and ipsilateral regions was similar. This is not the case in strong excitotoxic or stroke models, which can affect quantification [23].

When using the contralateral ROI, only the BP_ND[_^18^_F]GE-180_ was significantly higher than BP_ND-(R)-[_^11^_C]PK11195_, and as for core/contralateral ratio, BP_ND[_^18^_F]DPA-714_ showed a non-significant trend to increase (Table 2). When compared to the contralateral region, both a cerebellar input and a supervised clustering derived input produced significantly lower BP_ND_ values for all tracers. Using a cerebellar input showed no group differences between (R)-[^11^C]PK11195 and second-generation tracers in paired comparisons, while a cluster-derived input identified significantly lower BP_ND_ values with [^18^F]DPA-714 in animals dual-scanned with (R)-[^11^C]PK11195. The blood perfusion of the cerebellum in rodents is different than that in the brain and leads to a different tracer pharmacokinetic (i.e., a higher peak and uptake than in the normal brain/contralateral ROI, Fig. 3). It is also possible that the cerebellum contains some specific TSPO binding. Together, these two factors would lower the BP_ND_ values calculated in the core of the inflamed area by over-estimating the input of the reference tissue.

By the same rationale, a cluster-derived reference region could contain some specific binding, therefore introducing a bias; the MCAO model may not be optimal to define a reference cluster, as the ‘healthy’ tissue could itself contain a compartment of specifically bound tracer, further complicating the derivation of TSPO-free reference voxels in the LPS animal brains. However, this is unlikely since, in a comparison of the region of lowest uptake in control animals to the contralateral reference region in LPS animals, no significant difference in uptake was observed, supporting the use of the contralateral ROI as healthy reference tissue in this model. We applied this approach by reducing and reassigning the number of partitionable classes compared to the clinically established method, producing significantly lower BP_ND_ values for all tracers than a contralateral reference input to the SRTM. Clinical studies have used four or six class-supervised clustering; having also attempted to define four classes in the rodent brain (data not shown), we saw no obvious improvement to the BP_ND_ results from the LPS animals. This could be due to a number of factors: (*i*) unlike in the stroke model, the lower level of neuroinflammation induced in this LPS model may inherently not be separable into three or more kinetically distinct classes and/or (*ii*) the supervised clustering algorithm as it stands is not fit to use with preclinical images which have lower number of voxels to be used to identify classes.

Furthermore, [^18^F]DPA-714 BP_ND_ values were significantly lower than those from (R)-[^11^C]PK11195, while [^18^F]GE-180 results were not (Table 2). We speculate that this difference could be the result of voxels exhibiting pathology-related signal in LPS animals being mistakenly classed as normal tissue by the supervised clustering algorithm. In defining classes in the training data set, we were unable to identify a blood pool class such as that used in clinical supervised clustering [45], as the venous sinus in the rodent brain is smaller in diameter than the resolution of the PET camera, and external derivation (from, for example, the left ventricle of the heart) was not possible due to field of view limitations. The intermediate binding class defined instead, while reliably identified by the LMA brain segmentation, was kinetically not orthogonal to the normal tissue class. As these three radiotracers have similar affinities for TSPO, it is more likely that the supervised clustering algorithm mis-identified some ‘intermediate’ binding voxels as normal tissue leading to lower and slightly more variable BP_ND_ values for all tracers and hence the absence of significant differences between BPND for [^18^F]GE-180 and (R)-[^11^C]PK11195. Finally, if [^18^F]DPA-714 is more sensitive to these effects (i.e., if the voxels classed as normal tissue are more contaminated by TSPO-related signal, e.g., through inherently increased levels of microglial activation in these animals), it may explain the significant difference seen when estimating BP_ND_ with the supervised clustering approach.

Overall, these results suggest that (1) further work on the development and implementation of this method for preclinical imaging should be performed, and (2) the automatic segmentation of ROIs as implemented in the BrainVisa platform or PMOD® is currently the best approach, although unfortunately best suited for models with clearly identifiable rather than diffuse areas of neuroinflammation.

A recent study by Ory and colleagues using [^18^F]DPA-714 [34] validated the use of a reference tissue model with the contralateral striatum as input, producing BP_ND_ values which correlated well with a two-tissue compartment modelling approach. Interestingly, using a cerebellar input function to the SRTM in their high-dose LPS model produced BP_ND_ results which were also highly correlated with blood modelling, albeit with higher bias and producing BP_ND_ values lower than any other reference region, similar to what we observed in the present study. Discrepancies in these results, as well as those using the data-driven supervised clustering approach, which was unable to identify a suitable class of reference voxels, are most likely related to differences in the inflammatory responses induced by high (50 μg) and low dose (1 μg) of LPS. However, high doses of LPS inducing a neuroinflammatory response similar to what is observed after acute neurodegeneration are not representative of pathological conditions with lower neuroinflammatory levels, such as neurodegenerative diseases. Interestingly, the study also used a 90-min imaging window with this high-dose LPS injection; in the current study, we were able to show differences between tracers by the 60 min time point, suggesting that it is not necessary to scan for longer time periods with a low-dose LPS model or in a stroke model [17, 23]. Furthermore, it is well described in the TSPO imaging literature [49–51] that models or clinical cases in which a strong neuroinflammation (such as in stroke) are much easier to model consistently, whereas in the case of low or subtle neuroinflammation levels as observed in neurodegenerative diseases, the modelling is far more challenging, leading to discrepancies between studies. Considering the failure of the data-driven supervised clustering approach presented here, it is clear that further work is needed to determine a reliable reference tissue allowing the accurate quantification of TSPO tracer in preclinical studies without the need of performing arterial blood sampling when models of neurodegeneration such as transgenic mice or rats are used.

## Conclusions

The second-generation TSPO-PET radiotracer [^18^F]GE-180 showed an increased signal to noise ratio over (R)-[^11^C]PK11195 when considering dual scans whereas [^18^F]DPA-714 did not. However, the comparison of unpaired scans did not reveal such differences. The discrepancy between the statistical group results from paired and non-paired comparisons highlights the importance of performing dual scans for direct tracer comparison. Moreover, no significant differences were found between [^18^F]DPA-714 and [^18^F]GE-180. Altogether, these results suggest that when measuring low level of neuroinflammation, the differences between second-generation tracers (between them or when compared to (R)-[^11^C]PK11195) are small in term of sensitivity, while retaining their attractiveness in term of of-site use due to the longer F-18 half-life. Cerebellar and data-driven inputs to the SRTM were unable to produce comparable BP_ND_ values to those using a contralateral reference region. The choice of the contralateral ROI as reference tissue was supported by the fact that comparison between tracer uptakes in the contralateral ROI and healthy animals showed no significant difference and that *ex vivo* staining showed that the contralateral ROI contained very few activated microglia. Our study however also highlighted the remaining issue that when a low level of neuroinflammation present and no anatomically defined reference region can be identified, there is still no well-established method to identify a reference tissue in preclinical studies.

## Electronic supplementary material

ESM 1 (PDF 487 kb)
