# Supplementary material for: Comparative Evaluation of Three TSPO PET Radiotracers in a LPS-Induced Model of Mild Neuroinflammation in Rats
Source: Mol Imaging Biol. 2016 Aug 1;19(1):77–89. doi: 10.1007/s11307-016-0984-3 (PMC5209405; doi:10.1007/s11307-016-0984-3)
Supplement: Supplementary file 1 — (PDF 487 kb) [file 11307_2016_984_MOESM1_ESM.pdf]

Electronic Supplementary Material

Comparative evaluation of three TSPO PET radiotracers in a LPS-induced model of mild neuroinflammation in rats

Journal: Molecular Imaging and Biology

Sujata Sridharan[1], Francois-Xavier Lepelletier[1], William Trigg[2], Samuel Banister[3], Tristan Reekie[3], Michael Kassiou[3,4], Alexander Gerhard[1], Rainer Hinz[1], Hervé Boutin[1]*

[1] Wolfson Molecular Imaging Centre, University of Manchester, Manchester, UK.

[2] GE Healthcare, The Grove Centre, Amersham, Buckinghamshire, UK.

[3] School of Chemistry, University of Sydney, NSW 2006 Australia.

[4] Faculty of Health Sciences, University of Sydney, NSW 2006 Australia.

* Corresponding author: Wolfson Molecular Imaging Centre, University of Manchester, 27 Palatine Road, Manchester, M20 3LJ, UK.

Email: herve.boutin@manchester.ac.uk

Supplementary Table 1. Summary of injected doses, specific activities and injected amounts for each tracer (mean±SD).

Group	tracer	Injected dose (MBq)	Specific activity at injection (GBq/µmol)		Injected amount (nmol)	
			Mean	Range	Mean	Range
**1**	**$[^{11}C]$-(R)-PK11195$_{([^{18}F]GE\text{-}180)}$**	32.79±11.69	165.43±106.28	61.53-304.59	0.27±0.16	0.11-0.53
	**$[^{18}F]$GE-180**	29.26±5.35	149.92±98.13	68.58-344.13	0.24±0.10	0.09-0.35
**2**	**$[^{11}C]$-(R)-PK11195$_{([^{18}F]DPA\text{-}714)}$**	33.96±6.20	113.95±66.7	2.84-192.04	1.70±3.48	0.18-8.80
	**$[^{18}F]$DPA-714**	29.26±9.25	102.94±80.66	25.93-213.91	0.45±0.37	0.18-1.14
**3**	**$[^{18}F]$GE-180$_{only}$**	32.37±6.40	132.45±99.42	64.07-300.38	0.35±0.20	0.10-0.57
**4**	**$[^{18}F]$DPA-714$_{only}$**	34.77±6.91	58.57±40.98	13.41-109.53	1.00±0.82	0.34-2.22

Supplementary Table 2. Primary and secondary antibodies used for immunohistochemistry

Staining	Primary antibody, source (concentration)	Secondary antibody (concentration)
CD11b	Mouse anti-rat, AbD Serotec (1:1000)<br>Ref. Z0334	AlexaFluor® 594nm donkey anti-mouse (1:500)
GFAP	Rabbit anti-cow, Dako (1:400)<br>Ref. MCA275G	AlexaFluor® 488nm donkey anti-rabbit (1:500)
NeuN	Mouse anti-mouse, Chemicon (1:1000)<br>Ref. MAB377	AlexaFluor® 594nm donkey anti-mouse (1:500)
Claudin-5	Rabbit anti-Claudin-5, Sigma-Aldrich (1:100)<br>Ref. SAB4502981	AlexaFluor® 488nm donkey anti-rabbit (1:500)

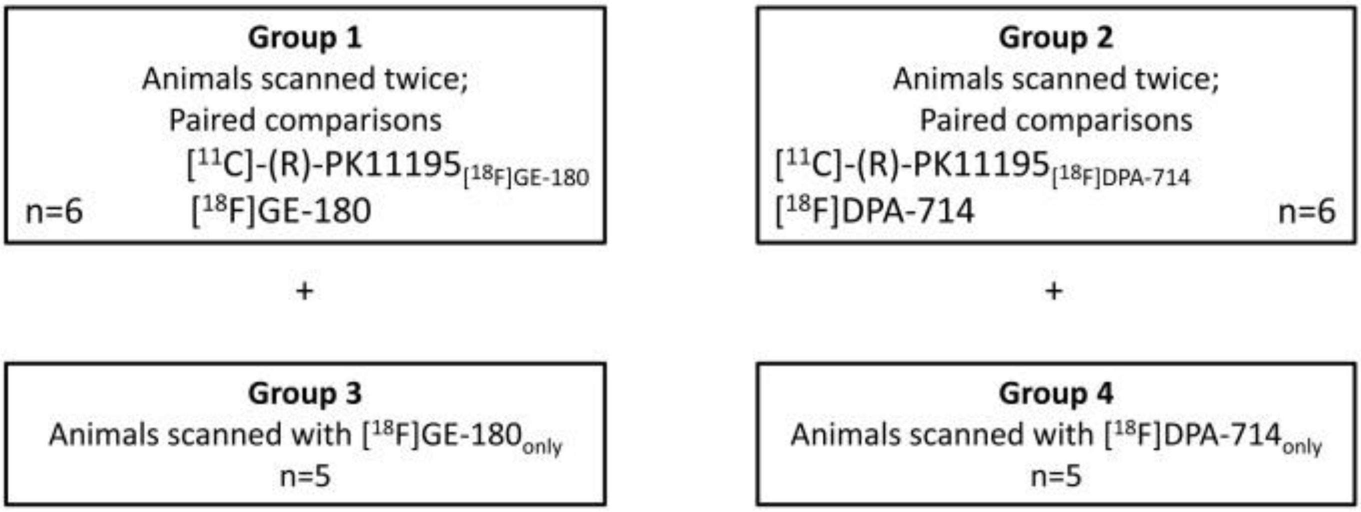

Supplementary Figure 1: Distribution of animal numbers per tracer and scanning group. Animals were dual-scanned with (R)-[^{11}C]PK11195 (n = 6) in the morning and [^{18}F]GE-180 (n = 6) in the afternoon or (R)-[^{11}C]PK11195 (n = 6) in the morning and [^{18}F]DPA-714 (n = 6) in the afternoon. A further n=5 animals each were scanned with [^{18}F]GE-180 or [^{18}F]DPA-714 only.

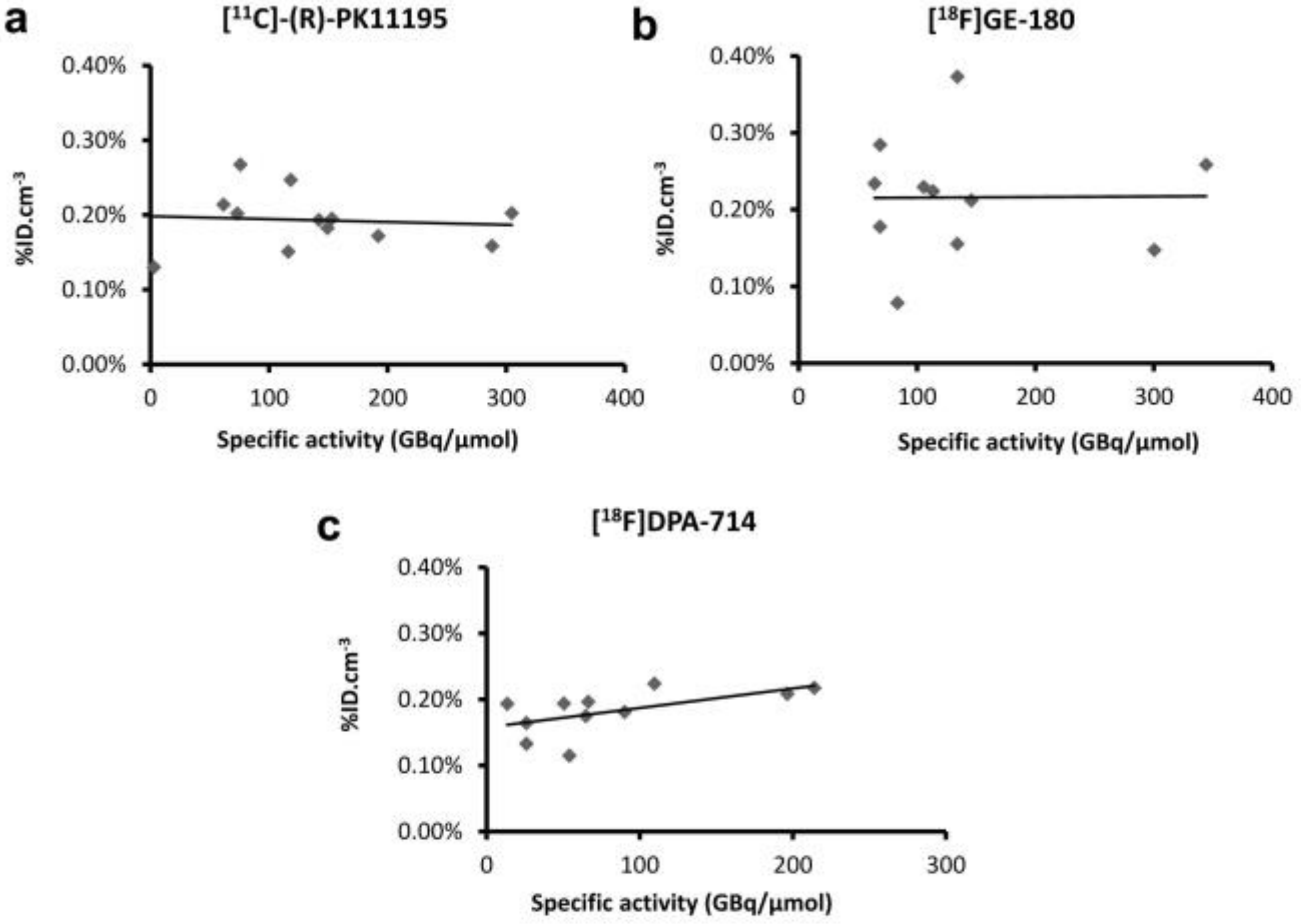

Supplementary Figure 2: Specific activity at injection time *vs.* % injected dose per cubic centimetre in the core of the LPS injection site for (**a**) (R)-[^{11}C]PK11195, (**b**) [^{18}F]GE-180 and (**c**) [^{18}F]DPA-714. r^2 values were, respectively, 0.01, 0.00 and 0.34; showing no relationship between the injected specific activity of each tracer and the corresponding uptake in the core of the LPS site.

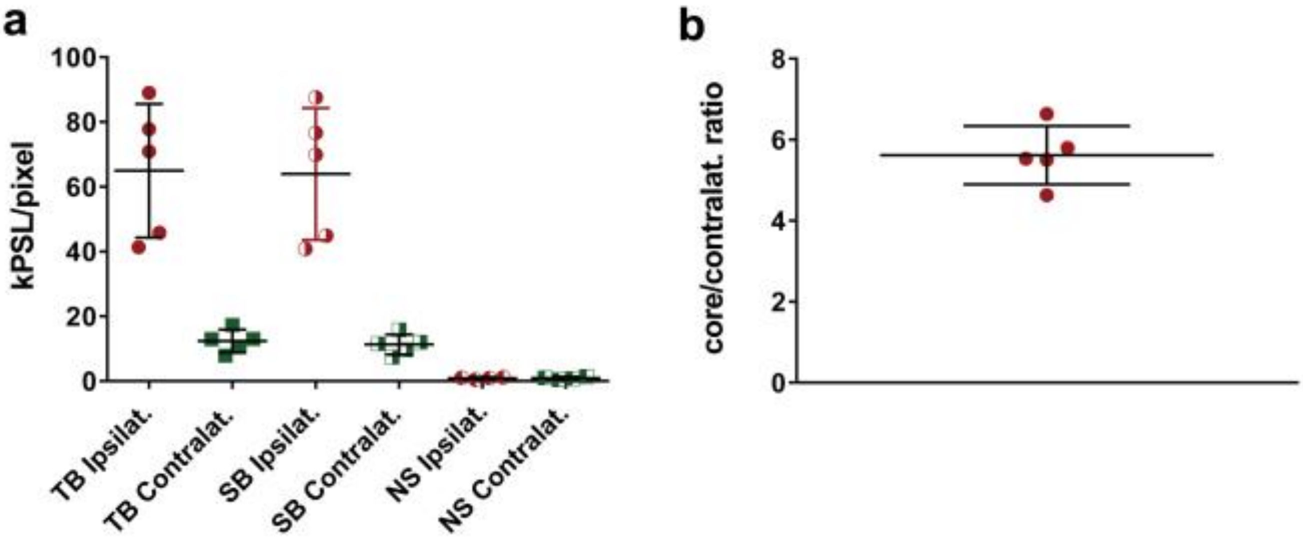

Supplementary Figure 3: (**a**) Quantification of the [^{18}F]DPA-714 autoradiography (TB: total binding (5nM [^{18}F]DPA-714), SB: specific binding, NS: non-specific binding access by co-incubation of 5nM of [^{18}F]DPA-714 with 5µM of PK11195) in the ipsilateral and contralateral striatum of rats 3 days after injection of 1µg of LPS in the right striatum and (**b**) core/contralateral ratio of the specific binding. Data are expressed as mean±SD of kPSL/pixel.
